# Advantages of inflatable multichannel endorectal applicator in the neo‐adjuvant treatment of patients with locally advanced rectal cancer with HDR brachytherapy

**DOI:** 10.1120/jacmp.v6i2.2029

**Published:** 2005-05-21

**Authors:** Slobodan Devic, Té Vuong, Belal Moftah

**Affiliations:** ^1^ Medical Physics Department McGill University Health Centre Montreal Quebec H3G 1A4 Canada; ^2^ Radiation Oncology Department McGill University Health Centre Montreal Quebec H3G 1A4 Canada

**Keywords:** endorectal, brachytherapy, dosimetry

## Abstract

High‐dose rate endorectal brachytherapy (HDR‐EBT) is mainly used as a palliative treatment modality. In this paper, we compare dosimetry distributions for a single‐channel catheter (Miami) applicator with distributions of the inflatable multichannel (Novi Sad) endorectal applicator. The comparisons were made with respect to dose coverage to the clinical tumor volume as well as to the bladder, rectal wall, prostate, and bone marrow. Our results suggest that a multichannel applicator provides better sparing of the bone marrow by 50%, clinically uninvolved parts of the rectal wall by 70%, and bladder and prostate (in the case of male patients) by 100% in terms of ratio of median doses to critical organ volume for single‐ and multichannel endorectal applicators. Our results justify the advantage of using a multichannel endorectal brachytherapy applicator as a neo‐adjuvant treatment of patients with locally advanced rectal cancer.

PACS numbers: 87.53.Jw, 87.53.Tf

## I. INTRODUCTION

High‐dose rate endorectal brachytherapy (HDR‐EBT) is mainly used as a palliative treatment modality in patients with locally advanced rectal cancer.^(1)^ In order to evolve toward a more radical treatment, a method that delivers conformal radiation to the rectal tumor bed while sparing the surrounding normal tissues is highly desirable. In 1998, we initiated a preoperative treatment protocol, based on an HDR‐ inflatable multichannel endorectal applicator for patients with operable, locally advanced rectal cancer. A high degree of the treatment conformity is desirable, to allow sparing of normal tissues and to improve the patient's quality of life after the treatment. The clinical aspects of the brachytherapy treatment technique were reported elsewhere.^(2,3)^

Palliative HDR‐EBT commonly uses a single‐channel rigid endorectal applicator that is associated with a significant amount of pain during the insertion, and therefore limits the clinical indications. The rectum curves at a distance of 10 cm from the anal verge, so the use of a rigid applicator (either single‐ or multichannel) is restricted to clinical indications of tumors below this curve. In this paper, we compare dosimetry distributions for a single‐channel catheter (Miami) applicator with distributions of the inflatable multichannel (Novi Sad) endorectal applicator. The aim of this study was to make a differential quantitative estimation of the expected dose distribution advantage for multichannel versus single‐channel endorectal applicators. The comparisons were made with respect to dose coverage to the clinical tumor volume as well as to surrounding critical structures, for example, bladder, rectal wall, prostate, and bone marrow.

## II. MATERIALS AND METHODS

**Figure 1:**
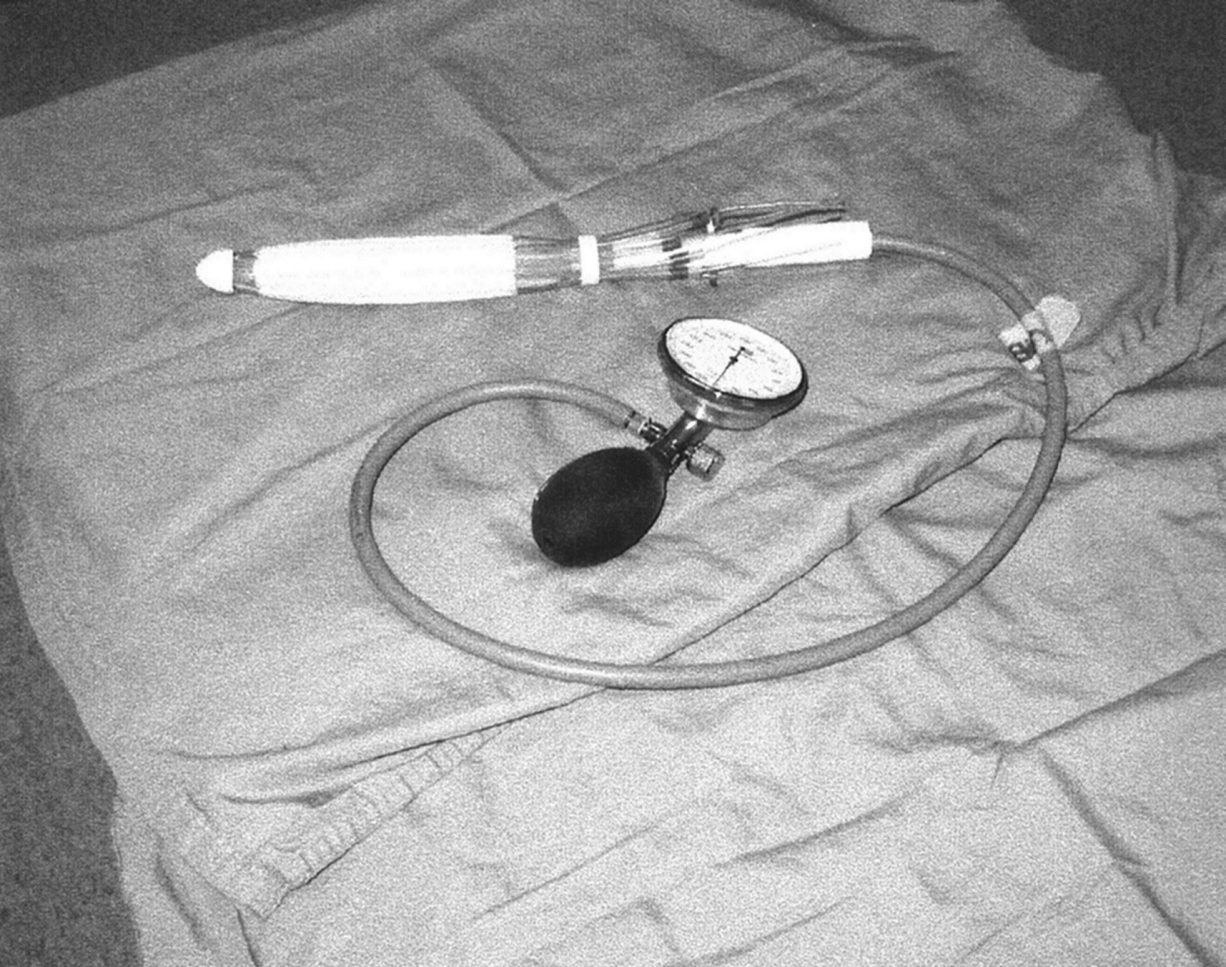
Inflatable multichannel endorectal brachytherapy applicator, Novi Sad (Nucletron)

In our department, patients with locally advanced rectal cancer were treated on protocol with preoperative HDR brachytherapy, using the Novi Sad (Novi) endorectal applicator (Nucletron Corp., Columbia, MD; Fig. 1). The Novi applicator consists of a central flexible tube with eight catheters arranged around the tube circumference. The applicator also contains a balloon‐type device, which can be inflated to immobilize the applicator in the desired position within the rectum. The target volume is localized using magnetic resonance imaging. Radio‐opaque endorectal clips are endoscopically inserted to mark the proximal and distal margins of the tumor. The treatment planning process commences at the CT simulator with the endorectal applicator adjusted according to the position of the radio‐opaque clips. Tumor and catheters are contoured and incorporated into digitally reconstructed radiographs. Digitally composite radiographs and 3D renderings are used to selectively enhance the visualization of bony landmarks as well as the inserted applicator and clips.

For treatment planning, catheters are loaded in a differential manner so that only those in a close proximity to the tumor contain active‐source dwell positions. This source‐positioning technique allows treatment of semi‐circumferential lesions in a conformal manner. Following the initial source position determination, a CT‐based brachytherapy treatment planning is performed to fully optimize the dose to the tumor, while limiting the dose to the immediate adjacent healthy tissues and the rectal wall. Dose distribution calculations were performed by a brachytherapy treatment‐planning system (Plato, v14.1, Nucletron, MD) for a sequence of CT planes. For 3D dose calculations, this treatment‐planning software uses the TG 43 protocol.^(4)^

**Figure 2:**
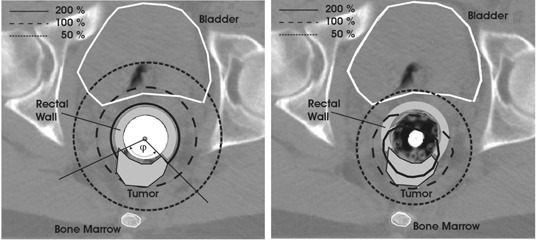
Comparison of dose distributions on the same slice for the rectum cancer treatment using a single‐channel (Miami, left) and multichannel (Novi, right) endorectal applicator. The slice shows three isodose lines: 50% (short dashes); 100% (long dashes); and 200% (solid line). The angle φ on the left the angular prevailing of the tumor volume around the rectum.

We made a quantitative comparison between plans created with the single‐channel (Miami) and multichannel (Novi) applicators. The plans were created so as to produce the same target dose‐volume histogram (DVH) curves for both plans, up to the level of the prescription dose, 650 cGy. For 27 patients (9 females, 18 males), we also calculated the DVH curves for the rectal wall, bladder, bone marrow, and prostate (for male subjects). This group of patients was chosen in such a way that the angular spread of the tumor, defined by an angle φ (Fig. 2, left), was not larger than 225° on at least two‐thirds of the CT slices that contained the outlined tumor volume. This selection was performed because the patients with circumferential lesions would not benefit if treated with a multichannel instead of a single‐channel endorectal applicator. The extent of the critical organs was determined in the following way: Each critical organ was initially outlined in its full extent. If the organ was encompassed within slices containing the target volume, the whole organ volume was used for DVH calculations. If, however, the organ extended beyond the level of the target volume, the superior and inferior limits of the critical organ in question were determined as the 10% isodose cloud spread throughout the irradiated volume. This was done with the intention of not biasing the data comparison with the anatomical differences between the patient cohort studied as well as the extent of the tumor within the 3D volume of the CT data. Also, the volume of rectal wall used for DVH calculations was calculated by subtracting the target volume from the outlined rectal wall.

In order to make a quantitative comparison between impacts of dose distributions on critical structure DVHs for the two applicators, we arbitrarily defined a parameter:

(1) $$\delta_{V50}=\frac{D_{Miami}(V=50\%)}{D_{Novi}(V=50\%)}$$

which represents a ratio of cumulative doses to a 50% of critical organ volume (median doses). The values D(V=50%) were read graphically from DVH curves.

## III. RESULTS AND DISCUSSION

Treated tumors were of an average depth of 1.8 cm (range 1 cm to 4 cm) and a mean length of 4.3 cm (range 2 cm to 10 cm). The longest distance of the tumor extent from the anal verge was 15 cm. During the course of treatment, all tumors were accessible by the Novi endorectal applicator, and conformal dosimetry was achievable in all cases.

**Figure 3:**
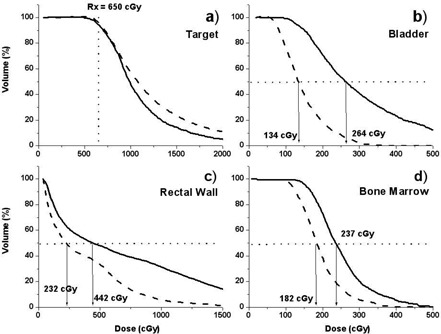
Cumulative DVHs calculated for the treatment plan (dashed lines, Novi applicator) and simulated plan (solid line, Miami single‐channel applicator) for one representative patient for (a) target volume, (b) bladder, (c) rectal wall, and (d) bone marrow.

Figure 2 represents a comparison of the 2D dose distributions obtained with the Novi applicator (right) and the single‐channel Miami applicator (left) on the same CT slice for one representative patient. For all patients, the same CT dataset was used for dose calculation for both Novi and Miami applicators. Positioning the source dwell positions in the middle of the Novi applicator simulated plans with the Miami applicator. Plans for the two applicators and every patient were made to produce the same tumor coverage, for example, an outlined target DVH overlap up to the prescription dose for both plans ((Fig. 3a). Figure 2 illustrates a better dose distribution conformity when the multichannel applicator is used versus a single‐channel endorectal applicator. It also produces a lower dose to the surrounding healthy tissues as well as to the clinically noninvolved part of the rectal wall, while the tumor coverage remains the same.

Figure 3 summarizes the DVHs calculated for one representative patient for the target volume as well as for the critical structures (bladder, rectal wall, and bone marrow) using the Miami (solid lines) and the Novi (dashed lines) brachytherapy rectum applicators. Dose‐volume histograms for the target volume ((Fig. 3a) illustrate results of the approach we used in order to provide the same dose coverage to the target volume for both the Novi and the Miami applicators. In addition, (Fig. 3a) indicates that a certain part of the target volume is receiving higher doses when using the Novi applicator. This is because the source dwell positions are closer to the target volume and because of the presence of higher dose gradients obtained with the Novi applicator. On the other hand, the conformal nature of the dose distributions produced with the Novi applicator lead to lower doses to the surrounding critical structures, observed on comparative DVHs.

From the DVH curves shown in Fig. 3 one may observe that critical structures will receive doses well below tolerance levels^(5)^ if the patient is treated with either of the two applicators. However, patients found to have positive nodes from pathological specimen after surgery would receive postoperative external beam radiotherapy (45 Gy/25 fractions) to the pelvic region.^(3)^

For these patients, improvement in dose conformity and reduction of dose to critical structures (as shown in Fig. 3) will facilitate external beam treatment planning with respect to critical organ dose tolerance restrictions.

**Figure 4:**
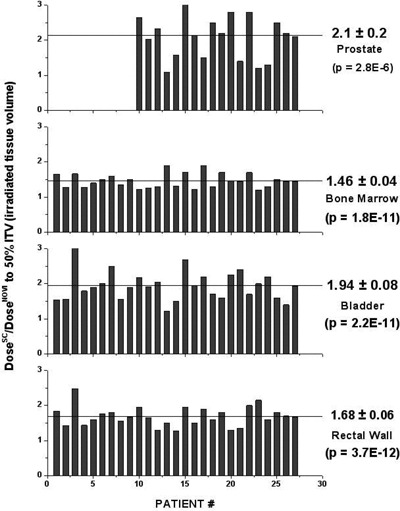
Ratio of median doses to the critical organ irradiated volume for single‐channel (Miami) and multichannel (Novi) applicator for the cohort of 27 patients. For 18 male patients the ratio was determined for the prostate. For every critical structure, we reported mean value of the ratio as well as the corresponding standard error and *p*‐values.

The ratio of $\delta_{v50}$ for the Miami and the Novi applicator for the cohort of 27 patients is presented in Fig. 4, together with the mean values as well as the corresponding standard errors. It is apparent from Fig. 4 that most dose sparing is obtained for bladder (even more for the prostate structure, but this applies for the male subjects only), while the least effect is observed in the bone marrow case. In the case of the prostate, a relative error of the average $\delta_{v50}$ ratio is 10% in comparison to 4% for the other three critical structures. Statistical significance of the mean for the $\delta_{v50}$ ratio has been calculated based on comparison with the same type of the distribution (having the same standard deviation) centered around the ratio value of 1, using the *t*‐type statistical test. Statistical significances for the ratios obtained in this way are given in parentheses in Fig. 4. Although well below the 0.05 value, statistical significance in the case of prostate is slightly higher than for other critical structures. This is due to the smaller number of patients in the distribution (males only), as well as the higher spread of the observed ratios around the mean.

Results of our numerical comparison, summarized in Fig. 4, have already been tested in phase I/II clinical trial in our department.^(3)^ It was observed that conformal preoperative HDR‐EBT for patients with locally advanced rectal carcinoma is well tolerated with Grade 2 moderate proctitis being the main toxicity.

Because of the central flexible tube in its current design, the Novi Sad applicator does not allow for the segmental shielding. However, flexibility of the Novi Sad applicator makes it more suitable for clinical applications by allowing its use at distances of more than 10 cm from the anal verge; it also provides more comfort to the patient.

## IV. CONCLUSIONS

The results of this study show that the multichannel brachytherapy endorectal applicator provides better conformity of dose delivery to the target volume while sparing the surrounding critical structures, when compared to the standard single‐channel endorectal brachytherapy applicator. The multichannel applicator provides better sparing of the bone marrow by 50%, clinically uninvolved parts of the rectal wall by 70%, and bladder and prostate (in the case of male patients) by 100% in terms of ratio of cumulative doses to a 50% of critical organ volume for single‐ and multichannel endorectal applicators. Therefore, the selection of a multichannel loading endorectal applicator is of importance in order to achieve conformal radiation delivery in the neo‐adjuvant treatment of patients with locally advanced rectal cancer with HDR brachytherapy.
